# Protocol for a mixed‐method systematic review on challenges perceived by final‐year undergraduate nursing students in a clinical learning environment

**DOI:** 10.1111/jan.14880

**Published:** 2021-05-24

**Authors:** Siti Hajar Ali, Nurul Husna Ahmad Rahman, Noorsuzana Mohd Shariff, Jalina Karim, Kok Yong Chin

**Affiliations:** ^1^ Department of Medical Education Faculty of Medicine Universiti Kebangsaan Malaysia Kuala Lumpur Malaysia; ^2^ Advanced Medical and Dental Institute Universiti Sains Malaysia Kepala Batas Malaysia; ^3^ Department of Nursing Faculty of Medicine Universiti Kebangsaan Malaysia Kuala Lumpur Malaysia; ^4^ Department of Pharmacology Faculty of Medicine Universiti Kebangsaan Malaysia Kuala Lumpur Malaysia

**Keywords:** challenges, clinical learning environment, clinical nursing education, degree nursing, final‐year, review protocol

## Abstract

**Aims:**

To determine the challenges perceived by final‐year nursing students in the clinical learning environment.

**Design:**

Data‐based convergent mixed‐method systematic review.

**Methods:**

Three electronic databases (Web of Science, Scopus, and Cumulative Index to Nursing and Allied Health Literature) will be used in the identification stage. The first search will use the search string for each database to identify relevant studies. The articles retrieved will be screened by year of publication, article type and language. Abstracts and full‐text of selected studies will be screened for eligibility independently by a minimum of two reviewers. The reference lists will be manually screened to identify additional publications. The quality assessment will be conducted by two reviewers using the Mixed Methods Appraisal Tools. Quantitative and mixed‐method studies will be transformed into qualitative. A thematic approach will be used to synthesize and report the data. Ethics approval and funding have been approved in April 2020.

**Discussion:**

This study will synthesize the types of challenges perceived by final‐year undergraduate nursing students in different clinical learning environments across the country.

**Impact:**

The proposed study findings will help nursing education stakeholders and faculty provide assistance to final‐year nursing students in their transition year to become registered nurses.

## 1 INTRODUCTION

In most countries, clinical nursing education contributes to more than 50% of the nursing curriculum (Arkan et al., 2018; Flott & Linden, 2016; Ibrahim et al., 2019; Jamshidi et al., 2016; Papastavrou et al., 2016). Nursing students must complete their clinical attachments to ensure competency and to become registered nurses. During the clinical attachments, students are required to apply academic knowledge and scientific skills along with professional attitude and values during patient care (Boyd‐Turner et al., 2016; Ibrahim et al., 2019; Midgley, 2006; Salizar & Nik Mohamed, 2016). Undergraduate nursing students are exposed to the clinical learning environment as early as their first semester. The study's duration and the development of the understanding of students in clinical learning affect how they perceive the clinical learning environment as a challenging area (Norfadzilah et al., 2018). As students complete their clinical nursing education, gradual interactions between the students and the elements in their clinical learning environments are expected to further create a sense of belongingness and prepare the students to become nurses (Ericson & Zimmerman, 2020; Ibrahim et al., 2019; Midgley, 2006).

## 2 BACKGROUND

Countries differ in nursing education. Undergraduate nursing education studies range in duration from 3 to 5 years (Anarado et al., 2016; Arkan et al., 2018; Sabatino et al., 2015). Final‐year undergraduate nursing students are expected to work independently as registered nurses after graduation because they have acquired the necessary knowledge and clinical experience through coursework and clinical attachments. Final‐year nursing students spend most of their credit hours in clinical settings and are familiar with the clinical learning environment. However, these students reportedly need to surmount many negative and positive challenges in their clinical learning environment during their final year of clinical attachments (Anarado et al., 2016; Atakro et al., 2019; Günay & Kılınç, 2018; Güner, 2015; Jamshidi et al., 2016). Previous literature indicates that the negative challenges may attenuate the students' motivation to pursue their nursing career (Makhlof & El‐Saman, 2017; Miligi et al., 2019; Shoqirat & Abu‐Qamar, 2013), thereby increasing the attrition rate and leading to a global shortage in nurses (Beitz, 2019; Ford et al., 2016; Liu et al., 2019; Lopez et al., 2018). Furthermore, ill‐prepared graduates will suffer from anxiety and stress due to incompetence and low confidence when facing real‐world challenges as professional nurses (Arkan et al., 2018; Bawadi et al., 2019; Ford et al., 2016; Sharif & Masoumi, 2005).

The literature on the challenges faced by final‐year undergraduate nursing students in the clinical learning environment is limited. A concept paper regarding factors that facilitate and inhibit clinical learning among undergraduate nursing students has been published (Mariyanti & Yeo, 2019). The article summarizes the studies published from 2003 to 2017; it shows that the theory‐practice gap is among the inhibiting factors and that a supportive clinical learning environment is one of the facilitating factors. A previous systematic review on barriers in clinical education from students', nurses' and lecturers' viewpoints in Iran is available (Shadadi et al., 2018). Four dimensions of obstacles, namely, individual, management, facilities and structures were mentioned; the article offers suggestions for resolving these obstacles. Despite the available evidence, the challenges perceived by final‐year nursing students in the clinical setting are still largely unexplored. Hence, a systematic examination of challenges from the perspective of the final‐year undergraduate nursing students is needed to enhance their learning experience and clinical competency.

## 3 THE REVIEW

### 3.1 Aim

The current review seeks to answer the following research question: What are the challenges perceived by final‐year undergraduate nursing students in the clinical learning environment? The formulation of the research question was guided by the “population, interest and context” (PICo) framework (Schardt et al., 2007). Population refers to undergraduate nursing students. Interest refers to challenges faced by the students. Context refers to the clinical learning environment.

### 3.2 Design/methodology

In view of this protocol being a mixed‐method systematic review and the data will be synthesized qualitatively, the authors modified the data synthesis part to accommodate qualitative data in accordance with Cochrane decision flowchart (Flemming et al., 2018). A data‐based convergent synthesis design will be used in this review. Data from the included studies (quantitative, qualitative and mixed‐method studies) will be analysed using thematic synthesis method. Quantitative data will be converted to a textual description (qualitizing quantitative data). This study will also use integrated design. Qualitized data from quantitative studies and mixed‐method studies, as well as qualitative data extracted from qualitative studies and mixed‐method studies will be integrated (Noyes et al., 2019). Integrated design aims to produce findings that can be readily synthesized into one another to answer the same review question (Noyes et al., 2019).

This is a systematic review, Preferred Reporting Items for Systematic Review and Meta‐Analysis Protocols (PRISMA‐P 2015) serves as a principal guideline in the development of this protocol (Shamseer et al., 2015). A few published mixed‐method systematic review protocols also adapt PRISMA‐P 2015 as their guidelines (Backman et al., 2018; Déry et al., 2019; Rose et al., 2016).

#### 3.2.1 Eligibility criteria

The initial screening will be performed by database filter according to the proposed inclusion and exclusion criteria. The inclusion criteria will include the following: studies published in the latest six years (between January 2015 and December 2020) to reflect the current clinical learning environment (Okoli, 2015; Xiao & Watson, 2017), which changes rapidly with the advancement in medical technologies; the decrease in the duration of patient's hospital stay; and learner's different needs within the clinical learning environment (Jaffe et al., 2019). Furthermore, only articles published in peer‐reviewed journals will be included to ensure the quality of the studies (Mohamed Shaffril, Ahmad, et al., 2020). Only articles published in English are selected to avoid misunderstanding of the content (Kitchenham & Charters, 2007; Mohamed Shaffril, Samsuddin, et al., 2020).

#### 3.2.2 Information sources

The literature search will be performed systematically using three databases, including Web of Science (WoS), Scopus and EBSCOhost CINAHL.

#### 3.2.3 Search strategy

Keywords used for the search will be based on synonyms and terms relevant to the research question. Alternate terms for ‘undergraduate nursing’, ‘challenges’ and ‘clinical placement’ will be used during the literature search to avoid missing eligible literature. These terms are based on previous studies on similar topics and suggestions by experts in the nursing field (Kitchenham & Charters, 2007). In the planning of the search strategy, an expert university librarian has been consulted (Kitchenham & Charters, 2007). The search string used in electronic databases is presented in Table 1. A reference list of included studies will be manually searched for the identification of additional articles.

**TABLE 1:** Search string

Database	Search string
WoS	TS = (("undergraduate nurs*" OR "baccalaureate nurs*" OR "degree nurs*") AND ("challeng*" OR "problem" OR "barrier" OR "hurdle" OR "obstacle*" OR "difficult*" OR “perception”) AND ("clinic*" OR "ward" OR "hospital" OR “medical cent*”))
Scopus	TITLE‐ABS‐KEY (("undergraduate nurs*" OR "baccalaureate nurs*" OR "degree nurs*") AND ("challeng*" OR "problem" OR "barrier" OR "hurdle" OR "obstacle*" OR "difficult*" OR “ perception”) AND ("clinic*" OR "ward" OR "hospital" OR “medical cent*”))
CINAHL	TITLE = (undergraduate nursing students OR nursing students OR student nurses AND challenges OR barriers OR difficulties OR issues OR problems OR limitations OR obstacles AND clinical) TITLE = (undergraduate nursing students OR nursing students OR student nurses AND challenges OR barriers OR difficulties OR issues OR problems OR limitations OR obstacles AND practice) TITL E= (undergraduate nursing students OR nursing students OR student nurses AND challenges OR barriers OR difficulties OR issues OR problems OR limitations OR obstacles AND clinical practice) TITLE = (undergraduate nursing students OR nursing students OR student nurses AND challenges OR barriers OR difficulties OR issues OR problems OR limitations OR obstacles AND in‐patients OR hospitalized patients) TITLE = (undergraduate nursing students OR nursing students OR student nurses AND challenges OR barriers OR difficulties OR issues OR problems OR limitations OR obstacles AND clinical practice OR clinical setting OR clinical placements)

#### 3.2.4 Data management and study selection

**FIGURE 1:**
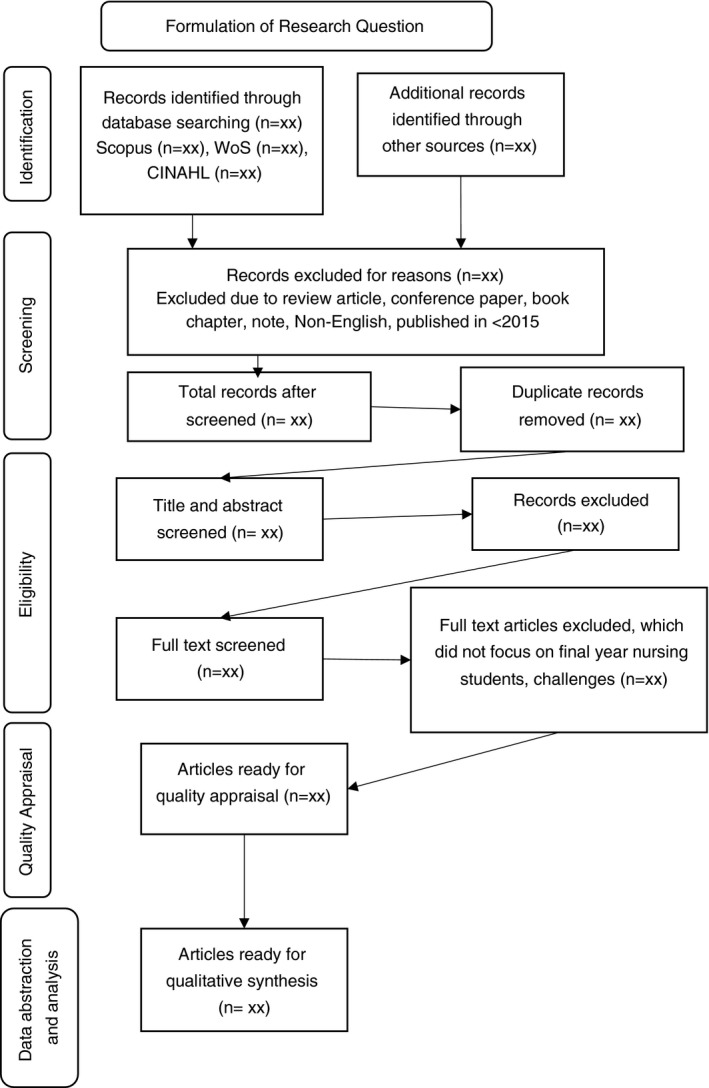
Flow diagram (adapted from Mohamed Shaffril et al., 2019)

Mendeley reference management software will be used to manage the data record and remove the duplicate articles. The second stage of screening will be restricted to the title and abstract of articles. At this stage, the titles and abstracts will be screened by a minimum of two reviewers to determine the eligibility of the articles. The final‐year undergraduate nursing students need to be the research subjects, and the challenges in the clinical setting need to be included. The universities have subscriptions to the three databases. Thus, access to articles is not restricted, and the full‐text can be downloaded. All potentially relevant full‐text articles will be read by at least two reviewers to further evaluate the article for review. If doubt persists, a third reviewer will be included for a consensus decision or majority vote. The reason for the exception will be noted. The process is shown in Figure 1.

#### 3.2.5 Data extraction

A minimum of two reviewers will extract the data independently. The data to be extracted are as follows: sample, study design, data collection method, country, clinical setting, results and recommendations. The data extraction template will be created using an Excel spreadsheet.

#### 3.2.6 Quality appraisal

The included articles will be reviewed by two reviewers independently. The Mixed Method Appraisal Tool (MMAT) 2018 (Hong et al., 2018) will be used to assess the methodological quality of the articles. The articles will be ranked qualitatively according to quality (low, moderate and high). Discrepancies between the reviewers will be discussed until a consensus is reached on whether the articles will be included or excluded for review. Only articles categorized as moderate and high quality will be selected for data extraction and synthesis.

#### 3.2.7 Synthesis of results (thematic synthesis)

This review will include qualitative data, considered as thick data, and quantitative data transformed into qualitative data, considered as thin data. The use of thematic synthesis is considered an appropriate approach in synthesizing both types of data that will further be integrated and can accommodate thin data (Booth et al., 2016). This review will follow Thomas and Harden's thematic synthesis to enable the authors to remain attached to key research results, synthesize them transparently and assist the production of new concepts or themes explicitly (Thomas & Harden, 2008).

The three stages of thematic synthesis include coding text, developing descriptive themes and generating analytical themes (Thomas & Harden, 2008). In stage one, the two reviewers will read the included articles to familiarize themselves with the articles. Then, findings will be read line by line and coded (inductive coding) to their meaning and content (creating initial codes) to answer the review question. Stage two involved generating descriptive themes from the initial codes. Similarities and differences between the codes will be viewed and further pooled in groups/themes that describe the challenges faced by final‐year nursing students. This process will be assisted by Atlas.ti. The codes and grouped codes can be visualized as network output in Atlas.ti. Finally, a discussion will be held among reviewers on any ideas, judgement and interpretations to develop analytical themes that can describe and/or explain all of the initial descriptive themes.

### 3.3 Ethical considerations

This systematic review is part of primary research conducted in a university. It has obtained ethical approval from the University Research Ethics Committee in April 2020 (Ethics reference number: UKMPPI/111/8/JEP‐2020‐270).

### 3.4 Validity and reliability/rigour

The systematic review protocol will be conducted in adherence to the Preferred Reporting Items for Systematic Reviews and Meta‐Analysis Protocols (PRISMA‐P). It will include the search strategy and database sources (Scopus, Web of Science, and CINAHL) used to perform the systematic review. The eligibility criteria will include inclusion and exclusion criteria, identification of relevant literature, quality assessment, data extraction and synthesis, and reporting (Moher et al., 2015). The PRISMA‐P 2015 allows the authors to publish the review protocol to ensure methodological rigour (Moher et al., 2015). Furthermore, a good documentation process ensures completeness and transparency, thus improving reliability because others can duplicate the study for cross‐checking and verification (Xiao & Watson, 2017).

## 4 DISCUSSION

Undergraduate nursing students experience different challenges in various study stages. Final‐year nursing students who have had more clinical exposure may perceive different challenges compared with freshmen and sophomores. However, the clinical learning environment must consider giving a clearer view of those challenges. This systematic review will extract the relevant articles from two leading databases and one nursing database to provide evidence‐based data regarding the challenges faced by final‐year nursing students in the clinical learning environment. Categories of challenges will be presented according to types, such as the physical structure of the clinical learning environment and interactions with individuals or groups. The study will also summarize the subjects' characteristics and clinical settings to elucidate the nature of the challenges.

### 4.1 Limitation

This review has potential limitations. The authors anticipate that the challenges will vary significantly across studies due to different nursing curricula and clinical settings experienced by final‐year undergraduate nursing students. However, authors will extract the data precisely according to country and clinical setting to allow the readers to compare the data in their context. Authors do not plan to extract personal problems, such as financial constraints and disabilities, because these factors are beyond the scope of the current study.

## 5 CONCLUSION

The challenges identified in this study will help nursing educators plan strategies to help students cope with the difficulties in the clinical learning environment and to motivate the students to further pursue their career in nursing (Boardman et al., 2019; Ericson & Zimmerman, 2020; Jamshidi et al., 2016). Recommendations to improve clinical learning will be put forward to the stakeholders and faculty to provide educational support systems that can equip nursing students with clinical competencies and to help enhance the students' clinical learning.

## CONFLICT OF INTEREST

The authors declare no conflict of interest.

## AUTHORS' CONTRIBUTION

SHA was responsible for the initial design of this study, developing and executing the draft search strategy and drafting the manuscript. NHAR was responsible for the initial design of this study, developing and executing the draft search strategy and revising the draft. NSMS developed and executed the draft search strategy and revised the draft. JK revised the draft. CKY developed and executed the draft search strategy and revised the draft. All authors have read and given final approval of the submitted manuscript.

### PEER REVIEW

The peer review history for this article is available at https://publons.com/publon/10.1111/jan.14880.

## Data Availability

Data available on request from the authors.
